# Ocular Blood Flow and Visual Function

**DOI:** 10.1155/2015/319254

**Published:** 2015-10-19

**Authors:** Goji Tomita, David Huang, Colm O'Brien, Ki Ho Park, Toru Nakazawa

**Affiliations:** ^1^Department of Ophthalmology, Ohashi Medical Center, Toho University, Tokyo 153-8515, Japan; ^2^Casey Eye Institute, Oregon Health & Science University, Portland, OR 97239, USA; ^3^Department of Ophthalmology, Mater Hospital, University College Dublin, Dublin 4, Ireland; ^4^Department of Ophthalmology, Seoul National University, Seoul 110-744, Republic of Korea; ^5^Department of Ophthalmology, Tohoku University, Sendai 908-8574, Japan

It is obvious that once blood supply to the retina, choroids, or optic nerve head is completely obstructed, severe visual functional damages occur. Even without such significant ocular blood flow insufficiency, ocular blood flow seems to influence developing or deteriorating glaucomatous damages as well as other retinal diseases. Recent advances in image analysis techniques have introduced new algorithms to study the retinal circulatory disturbances and the mechanisms underlying their pathology. Knowledge and understanding of these conditions may lead to successful therapies and provide better care to patients. For this purpose the continuing efforts to understand the role of ocular blood flow for developing retinal and optic nerve head pathology, the development of strategies to treat these conditions, and the evaluation of outcomes are important.

Last year, we invited investigators to contribute original research as well as review articles that address this field. We encourage manuscripts that will describe the new developments of technologies for evaluating ocular blood flow, new insights into relationship between ocular circulatory disturbances and visual function, blood flow and structure relationship, effects of intervention for ocular blood flow on disease progression, and current concepts in ocular blood flow and its relationship to extraocular determinants influencing blood vessel itself or those of systemic factors such as aging, nocturnal hypopressure, or cardiovascular disease. Today, we are very happy to publish this special issue of this journal.

In this issue, original research and review articles are accepted. The research papers focused on using color Doppler imaging techniques to understand relationship between retrobulbar hemodynamics and visual field progression in normal tension glaucoma, evaluating differences in the vascular response to a hypercapnic stimulus between normal tension glaucoma patients and normal subjects, investigating the role of systemic arterial stiffness evaluated using brachial-ankle pulse wave velocity in glaucoma patients with diabetes mellitus, and comparing the ocular pulse amplitude lowering effects of preservative-free tafluprost and dorzolamide-timolol fixed combination using dynamic contour tonometry. Besides those of analyzing glaucoma and blood flow relationship, the research papers dealing with detecting evidence for retinal damage evolving from reduced retinal blood flow in carotid artery disease, evaluating the effects of long-term tamponade with silicone oil on retinal saturation, and evaluating reproducibility of ocular circulation measurements using laser speckle flowgraphy in neonates are involved.

The review described the present status of understanding relationship between ocular blood flow and glaucoma, including advancements in noninvasive imaging technologies; those have led to the characterization of magnitude and time course in retinal blood flow response to stimuli, describing systemic and ocular hemodynamic risk factors in glaucoma, and role of ocular blood flow in developing normal tension glaucoma. The papers sample the extraordinary progress that has been made in the field.

Current research suggests that new developments will continue to follow. Particular importance will be given to manuscripts that address the development of techniques for evaluating systemic and ocular blood flow as well as those adopting such techniques for understanding blood flow and disease relationships. Our aim is to shed light on important knowledge gaps and to provoke thoughts for further research and the development of therapeutic strategies.

*Goji Tomita* *David Huang* *Colm O'Brien* *Ki Ho Park* *Toru Nakazawa*

